# MRI-compatible abdomen phantom to mimic respiratory-triggered organ movement while performing needle-based interventions

**DOI:** 10.1007/s11548-024-03188-x

**Published:** 2024-06-05

**Authors:** Ivan Vogt, Katja Engel, Anton Schlünz, Robert Kowal, Bennet Hensen, Marcel Gutberlet, Frank Wacker, Georg Rose

**Affiliations:** 1 Research Campus STIMULATE, Otto von Guericke University, Magdeburg, Germany; 2 Faculty of Electrical Engineering and Information Technology, Otto von Guericke University, Magdeburg, Germany; 3 Institute of Diagnostics and Interventional Radiology, Hannover Medical School, Hannover, Germany

**Keywords:** Motion phantom, Organ motion simulation, PVA cryogel, MRI-compatible actuator, Needle-based interventions, Image-guided interventions

## Abstract

**Purpose:**

In vivo studies are often required to prove the functionality and safety of medical devices. Clinical trials are costly and complex, adding to ethical scrutiny of animal testing. Anthropomorphic phantoms with versatile functionalities can overcome these issues with regard to medical education or an effective development of assistance systems during image-guided interventions (e.g., robotics, navigation/registration algorithms). In this work, an MRI-compatible and customizable motion phantom is presented to mimic respiratory-triggered organ movement as well as human anatomy.

**Methods:**

For this purpose, polyvinyl alcohol cryogel (PVA-C) was the foundation for muscles, liver, kidneys, tumors, and remaining abdominal tissue in different sizes of the abdominal phantom body (APB) with the ability to mimic human tissue in various properties. In addition, a semi-flexible rib cage was 3D-printed. The motion unit (MU) with an electromagnetically shielded stepper motor and mechanical extensions simulated a respiration pattern to move the APB.

**Results:**

Each compartment of the APB complied the relaxation times, dielectricity, and elasticity of human tissue. It showed resistance against mold and provided a resealable behavior after needle punctures. During long-term storage, the APB had a weight loss of 2.3%, followed by changes to relaxation times of 9.3% and elasticity up to 79%. The MU was able to physiologically appropriately mimic the organ displacement without reducing the MRI quality.

**Conclusion:**

This work presents a novel modularizable and low-cost PVA-C based APB to mimic fundamental organ motion. Beside a further organ motion analysis, an optimization of APB’s chemical composition is needed to ensure a realistic motion simulation and reproducible long-term use. This phantom enhances diverse and varied training environments for prospective physicians as well as effective R&D of medical devices with the possibility to reduce in vivo experiments.

## Introduction

In recent years, the medical device market experienced substantial growth, with global revenues reaching US$490bn [1]. To ensure the safety and effectiveness of medical technology, it is subject to strict regulations such as the ISO 10993-1 and ISO 14155 which highlight the need for in vivo testing. However, clinical trials are costly and complex, adding to ethical scrutiny of animal testing [2]. For this reason, there is a growing need to promote alternatives to in vivo testing, such as the Center for Alternatives to Animal Testing (CAAT). This demand leads to an increase in publications in the field of phantom development, addressing diverse applications such as surgical training and the validation of software and hardware for medical device development [3].

Nowadays, image-guided interventions are becoming increasingly important as they allow short recovery times and thus improved patient outcome. Compared to other clinical imaging modalities, magnetic resonance imaging (MRI) offers an incomparable soft tissue contrast, a radiation-free examination, noninvasive temperature measurement, and multiplanar imaging, enabling spatial tracking of the interventional instruments. However, there are drawbacks such as limited patient access due to the very confined space in the MRI bore, the need of MRI-compatible tools due to the strong magnetic fields, and the sensitivity of MRI to external electrical devices during interventional MRI (iMRI). [4]

These highlight the need to develop new tools to be tested under in vivo conditions (e.g. under patient breathing) to ensure the safety and effectiveness during iMRI. Since animal or patient trials should be avoided, suitable and realistic phantom studies are crucial. So far, phantoms for research and commercial issues were designed for only few specific aspects, whether it is the accurate imaging of human anatomy for the chosen imaging modality [5–8], the precise movement of organs during respiration [9–12] or a realistic puncture impression [7, 8, 13].

To overcome the current challenges, this work presents an affordable and customizable abdominal motion phantom to simulate MRI properties, the human anatomy, and the respiratory-triggered organ movement for needle-based interventions.

## Materials and methods

### Abdomen phantom body (APB)

**Fig. 1:**
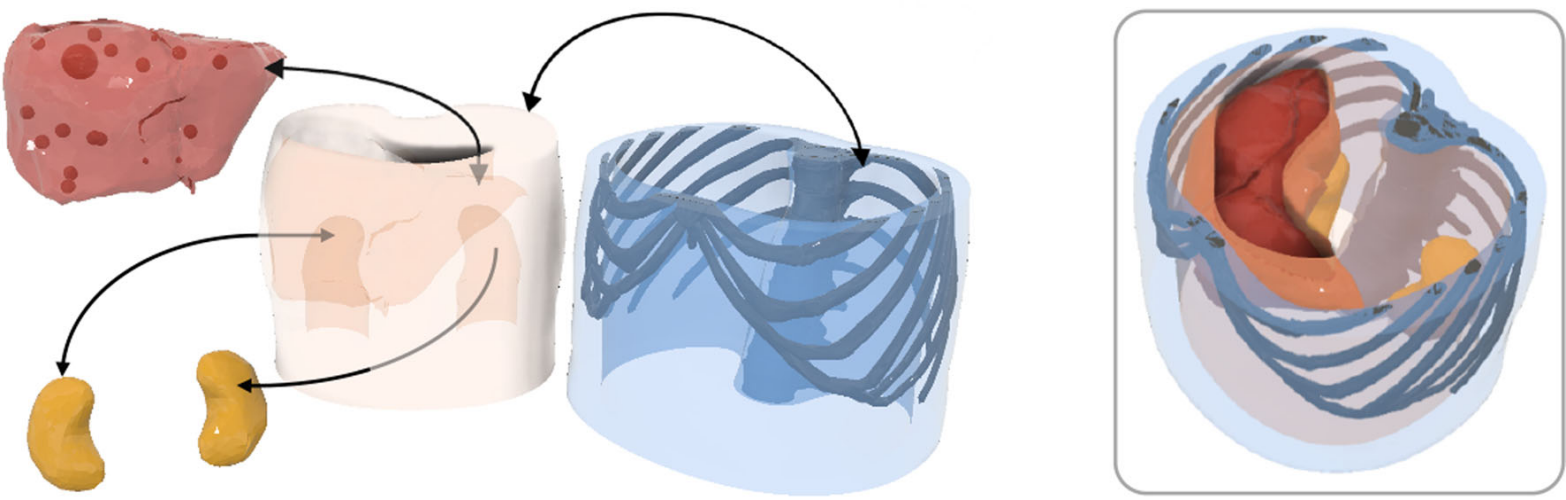
The APB consists of separately fabricated parts: (blue) muscle and rib cage; (orange) MAT; (yellow) kidneys; (red) liver including tumors (1 × Ø3 cm, 13 × Ø1.5 cm, 3 × Ø5 mm)

The abdomen phantom body (APB), based on segmented CT-scans [14], consisted of muscles, merged abdominal tissue (MAT: stomach, spleen, intestine), rib cage, liver, liver tumors and kidneys to mimic the main abdominal anatomy (see Fig. 1). Thereby, the soft tissue was manufactured using polyvinyl alcohol (PVA, KurarayPoval 15-99, Kuraray Europe GmbH) which becomes a cryogel (PVA-C) after at least one freeze–thaw cycle (FTC). The FTCs cause physical cross-linking in the PVA and generate the solid cryogel from the liquid. Prior research has shown biocompatibility, low production costs, elasticity, and lubricity of this unique gel with the potential to be an ideal phantom material to mimic soft tissue [15]. By varying the concentration and the FTC of PVA as well as adding sugar (refined sugar, REWE Markt GmbH) [16] sodium chloride (NaCl, table salt, REWE Markt GmbH) [16], and potassium sorbate (PS, E202, Buxtrade GmbH) [17], not only the mechanical but also MRI relevant properties can be customized. In addition, PS supported in preventing mold formation.

To model the rib cage associated with the abdomen, the ribs and their vertebral bodies (Th10-L4) were 3D-printed using polycarbonate (PC, Stratasys Inc.), a suitable material for bones [18]. Using the same method, the rib cartilages were made of flexible thermoplastic polyurethane (Flexfill TPU 98A, Fillamentum Manufacturing Czech s.r.o.) to ensure elasticity of the rib cage during respiration simulation. All parts were assembled with flexible superglue (Turbo Fix^2^ Flex, UHU GmbH & Co. KG).

The following compartment compositions (see Table 1) were iteratively determined until a high agreement of the APB`s properties with the real tissue parameters was achieved.

**Table 1:** FTC with freezing time (Ft), thaw time (Tt), and mass concentrations of the substances of each compartment. The tumors were additionally dried 3 days at 21 °C

Compartment	FTC/Ft at − 20 °C /Tt at 21 °C	Concentration [m%]			
		PVA	PS	Sugar	NaCl
Muscles	3/15 h /18 h	12	1	20	0.5
MAT	2/24 h /24 h	15	5	0	0
Liver	2/15 h /15 h	13	0.5	35	0.6
Liver tumor	5/13 h /15 h	12	0.5	0	0.5
Kidneys	4/12 h /12 h	12	0.5	0	0.5

To address diverse applications, the APB’s design allows the replacement of the liver and kidneys, without the manufacturing of an entirely new phantom. Hence, alternative phantom materials or even real organs can be inserted.

### Motion unit (MU)

**Fig. 2:**
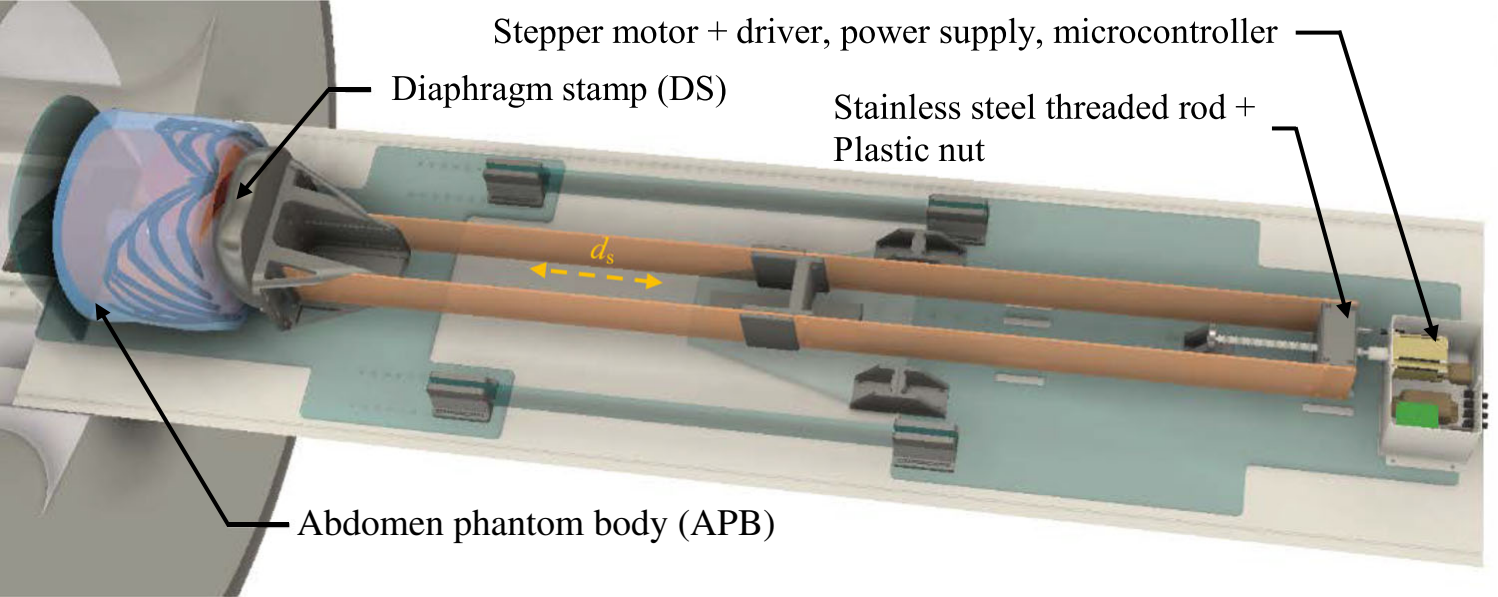
The MU consists of static acrylic plates (turquois), moving acrylic plates (orange) and the drive unit (white) to deform the APB with the DS. (gray) 3D-printed PETG parts as connectors

One of the crucial requirements of the motion unit (MU) is MRI compatibility and safety. Typically, MRI-compatible pneumatic, hydraulic or piezoelectric actuators were used. However, these approaches result in expensive and self-made solutions with a significant implementation effort [19]. Consequently, a NEMA 23 stepper motor (23HE30-2804S, StepperOnline) was utilized to drive the MU (see Fig. 2). Here, an aluminum threaded rod and nut (DST-LS-12X25, igus® GmbH) was used to create variability and precision for the breathing pattern. The motor was controlled by a driver (TMC 5160 StepStick, TRINAMIC Motion Control GmbH), a microcontroller (Arduino® NANO), and a power supply (IRM-30-24ST, MEAN WELL Inc.). All electronics were filtered and housed in an aluminum enclosure (517-4156, RS Components GmbH). Additionally, the drive unit was placed at a distance of 190 cm from the APB via linear extensions to ensure MRI compatibility and safety. The mechanical elements were made of acrylic glass (XT_5mm, Kunststoffplattenonline GmbH), connected with brass screws and 3D-printed polyethylene terephthalate glycol (PETG, Fillamentum Manufacturing Czech s.r.o.) parts.

The diaphragm inspired stamp (DS) executed the breathing patterns for the specific organ movement with a expansion *d*_s_ (typical range of *d*_s_ is 1.5–3.5 cm [20]) by compressing the inner part of the APB. Thereby, the position *s*(t) within the breathing cycle over time was defined by

1 $$  s\left( t \right) = \frac{{d_{s} }}{2}\left( {{\text{cos}}\left( {\frac{{t \cdot \pi }}{T}} \right) + 1} \right);\;T = \left\{ {\begin{array}{*{20}l}    {T_{i} } \hfill & {\rm{for}\; - T_{i}  \le t \le 0} \hfill  \\    {T_{e} } \hfill & {\rm{for}\;0 < t < T_{e} } \hfill  \\   \end{array} } \right. $$

The inhalation time *T*_i_ and the exhalation time *T*_e_ can be individually set to a desired physiological breathing frequency *f*_b_ (typical range of *f*_b_ is 14–19 mm^−1^ [21, 22]).

### Manufacturing of the abdomen phantom

Distilled water and PVA granulates were heated in a stainless-steel container at 96 °C with constant stirring until all granulates swelled and dissolved, forming a viscous transparent liquid. To maintain the desired concentration any mass difference after boiling was compensated with distilled water. NaCl was added during boiling, while sugar and PS were introduced post-boiling. All concentration were expressed in mass percentages (m%).

**Fig. 3:**
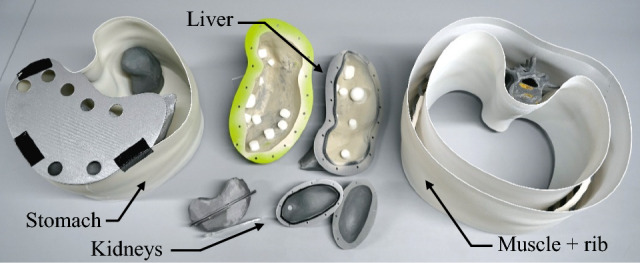
Manufacturing molds. The stomach area features placeholders for liver and kidneys. The liver tumors are glued with PVA on the liver mold

After the solutions had cooled down to room temperature, the molds (Fig. 3) were filled and sealed to produce separated APB parts (Fig. 1). Beforehand, the dried liver tumors were manufactured in silicon molds (014257, 013656, 008820, MAE SARL) and glued onto the liver mold with a non-frozen solution of the identical substance, acting as an adhesive. The freeze–thaw cycles were completed in an industrial freezer according to the pre-selected duration and cycles (see Table 1).

The manufacturing time of the compartments ranged between 4 and 6 days including preparation of the solutions, molding, and FTCs.

### Experiments

**Fig. 4:**
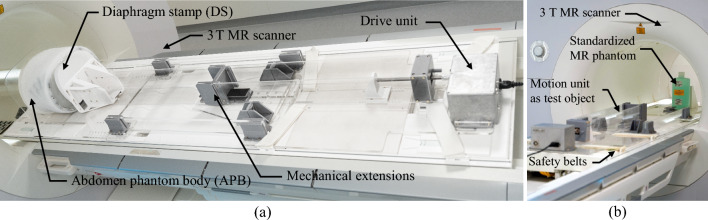
**a** Proposed experimental setup of the motion phantom within an MRI environment. For safety reasons belts are used to fixate the drive side on the MRI table. **b** Setup to analyze the MR-compatibility of the MU

To prove the versatile functionalities within the MRI environment, the MRI relevant properties (relaxation times, dielectricity), the mechanical performance, and the durability of the APB were analyzed [23]. Furthermore, the APB and the MU were combined to assess the MRI compatibility and reliability of the organ motion simulation. The test environment was a 3 T MRI system (Siemens MAGNETOM Skyra, Siemens Healthineers AG), as shown in Fig. 4a. For image analysis as well as data processing ImageJ [24] and MATLAB (The MathWorks Inc., version 9.9.0 (R2020b)) were used.

#### MRI properties

The relative tissue intensity in MRI is significantly dependent on relaxation times of individual materials [25]. To analyze the MRI properties of the APB, the following sequences were performed using a 4-chanel multipurpose coil and a spine coil (Flex Large 4, Spine 32, Siemens Healthineers AG) [26, 27]: MPRAGE sequence for T1, TR = 7 s, TE = 3 ms, voxel size = 2 × 2 × 4 mm, TI: 516–6500 ms; SE sequence for T2, TR = 5 s, voxel size = 2 × 2 × 3 mm, TE: 12–1000 ms. Additionally, the loading behavior of the radio-frequency (RF) coils is known to affect image contrast and signal-to-noise-ratio (SNR), which can be manipulated by the dielectricity of materials [28]. The APB’s permittivities ε_r_ and loss tangents tan δ for 3 T were measured with the coaxial probe method [16] by using a dielectric probe and a vector network analyzer (N1501A and N9923, Keysight Technologies).

#### Mechanical properties

To create a suitable organ movement and needle puncturability, the APB should match the mechanical properties of the human tissue. The determination of the elasticity *E* of the respective compartment, compression tests according to ISO 604:2003 were performed with a testing machine (zwickiLine Z0.5 TN, ZwickRoell GmbH). Thereby, a cylindrical probe (35 × 22 mm) was preloaded to 0.1 N and compressed 10 times with a speed of 10 mm/min. The Young’s modulus was assessed via the secant module method at 5% and 10% according to [29].

#### Durability

The long-term tests provided an assessment of the durability of each compartment during regular use based on certain criteria. Each cylindrical sample (47 × 29 mm) was stored in an airtight box at room temperature.

The weight was measured with a precision scale and visually inspected for mold at weekly intervals for three months. Representatively, a liver sample was punctured with a needle (KIM-18/15 T, innotom GmbH) 200 times in a Ø 40 mm area. The imaging as well as mechanical properties of each sample were compared with the initial values. Furthermore, a combined liver-MAT sample was compressed 153,000 times with 30 N over 5 days to approximately simulate one week of high continuous loading at *f*_b_ = 14 min^−1^. After this test, the elasticity was analyzed again.

#### MRI compatibility

Investigations are necessary to determine whether RF signals potentially emitted by the electrically-driven MU affect MR image quality in terms of artifacts and/or SNR degradation in the scanner room. According to IEC 62464-1, a standardized MR phantom (1900 ml, cylindrical, 8,624,186 K2285, Siemens Healthineers AG) instead of APB was scanned in sagittal (sag), transversal (tra), and coronal (cor) orientations using spin-echo (SE) imaging (see Fig. 4b). The SNR was compared between the two states: reference (no MU) and active (motor on).

#### Organ movement

The APB was scanned with a T1 w VIBE volume (TR = 4.5 ms, TE = 1.3 ms, voxel size = 1 × 1 x 1 mm) [30] at paused d_s_{0,3} inhale (*T*_i_ = 1.3 s) and exhale (*T*_e_ = 2.3 s) states 10 times to compare organ displacement with human data [21]. The spatial positions of several representative landmark points of the liver and each kidney in MR images were evaluated in each breath-hold state. Additionally, the reproducibility of the displacement was analyzed.

## Results and discussion

### MRI and elasticity properties

**Fig. 5:**
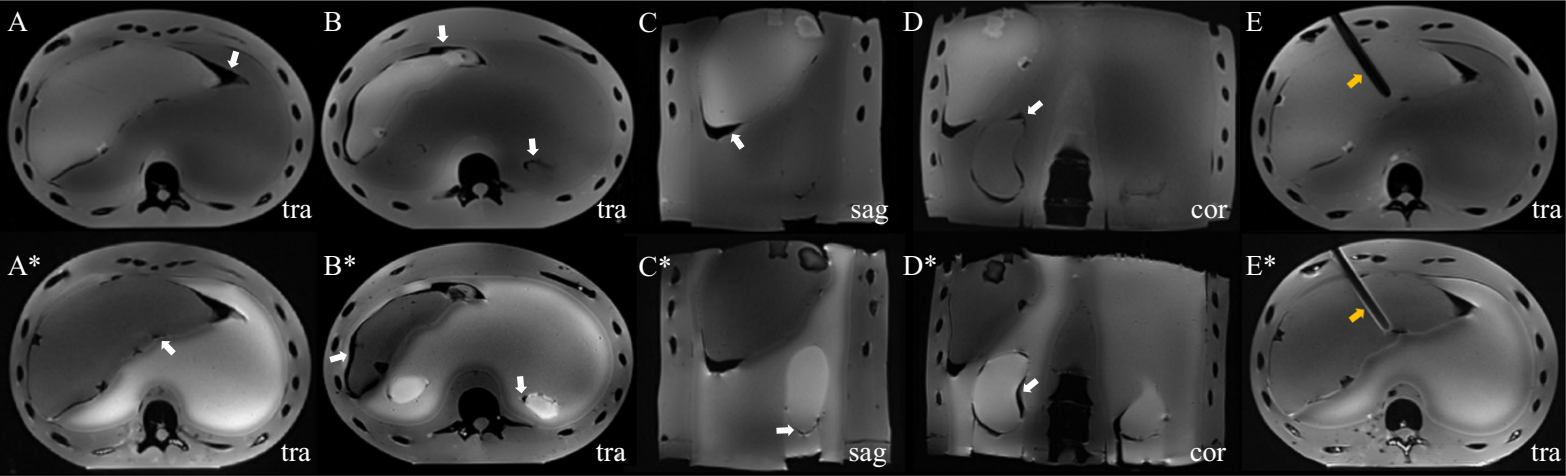
MRI of the APB in transversal (tra), sagittal (sag), and coronal (cor) views. (A-E) T1-w VIBE (TR = 4.5 ms, TE = 1.3 ms, voxel size = 1.5 × 1.5 x 1.5 mm) 30. (A*-E*) T2-w TSE (TR = 2200 ms, TE = 97 ms, voxel size = 1 × 1 × 3 mm) 31**.** (White arrows) air gaps/holes. (Orange arrows) Intended artifact of an inserted needle

The achieved T1/T2 and Young’s modulus values of the APB are within the broad range of literature values for human tissue (see Table 2). In contrast, the APB’s dielectric properties indicated an average deviation of 13.2% and a maximum deviation of 30% compared to human values. Figure 5 shows the intended anatomical structure, but with unintended air gaps around each organ group caused by non-optimal mold sizes of the separately fabricated compartments. Since most air gaps remain below 1 mm in diameter, the haptic experience is not affected during interventional needle insertions, while the largest air gaps around the liver (marked in Fig. 5 A, C) may cause misinterpretation and inaccurate needle positioning. However, in typical clinical liver interventions, these areas are not part of the needle path. The real property values of the MAT were averaged which allows a wide variability of the relative intensity to other organs by changing the substances’ concentrations. The intended tumors were hypointense on T2-w and slightly hyperintense on T1-w images, reflecting clinical cases of hepatic adenomas or fat containing liver metastases [31]. Due to the APB’s manufacturing process, a variation of MRI and mechanical properties is to be expected. By optimized molds, its sizes, and fabrication of PVA-C the presented inaccuracies can be further minimized.

**Table 2:** Comparison of MRI and mechanical properties of the APB (mean ± standard deviation) with {real tissue [26, 27, 32–40]}. Long-term results to estimate the durability of each compartment

Sample properties	Muscles	MAT	Liver	Kidneys
T1 [ms]	840 ± 32 {865–1659}	1070 ± 20 {377–1328}	699 ± 28 {680–977}	1183 ± 31 {988–1907}
T2 [ms]	29 ± 1 {27–53}	68 ± 2 {36–97}	29 ± 1 {25–38}	89 ± 3 {37–89}
*ε*_r_	72.9 {63.9}	83.4 {67–89}	66.4 {64.96}	79.2 {90.9}
tan *δ*	1.2 {1.6}	2.4 {0.9–2.8}	0.8 {1.1}	1.6 {1.4}
*E* [kPa]	53.7 ± 0.1 {19.3–2200}	19.74 ± 0.02 {1.2–46}	32.2 ± 0.3 {2.2–386}	86.63 ± 0.2 {86–4060}
Weight loss [%]	7.4	0.8	5.1	2.5
T1/T2 change [%]	− 9.5/− 10.3	6.5/12.7	− 10.2/− 9.1	− 8.9/− 6.5
*ε*_r_/tan *δ* change [%]	− 0.1/12.3	− 4.5/− 11.3	− 6.1/− 5.1	− 2.2/− 0.4
*E* change [%]	47.4	61	78.7	21.2

### Durability

**Fig. 6:**
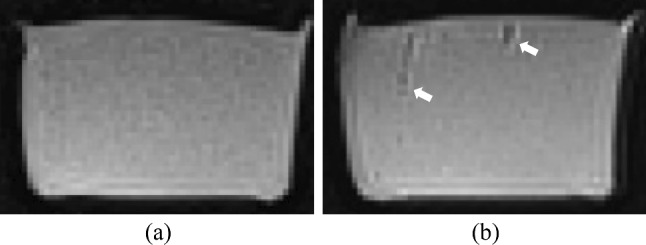
SE imaging (TR = 2000 ms, TE = 25 ms, voxel size = 0.9 × 0.9 × 0.9 mm) of a liver sample after 100 (top) and 200 (bottom) needle punctures. White arrows indicate air gaps/holes

The storage test results demonstrated the resilience of the sample compartment against mold, showing a weight decrease of approximately 2.3%. Notably, 98% of this weight loss occurred within the initial 14 days. Thereby, the MRI relevant properties were changed on average by 7.2%. Based on high elasticity changes during storage, the long-term reproducibility while mechanical testing with the APB is not provided. After 100 needle punctures, tissue resealability was still observed, but also a slight decrease in elasticity of 1.2% (see Fig. 6). The insertion holes were visible after 200 punctures in high resolution MRI (0.9 mm isotropic) followed by an elasticity decrease of 2.8%. After the long-term compression, the MAT-liver sample had deformed plastically and lost 12.5% in height as well as 10.5% of its elasticity. Since, the testing extended over several days, a storage-related property change can occur additionally to the compression impact.

The long-term stability of APB is affected by changes in crystallinity and pore structure caused by factors such as FTCs. These alterations impact diffusion behavior, possibly leading to unintended water diffusion, which has a direct influence on the imaging and mechanical properties of APB [15]. Thus, future research into stabilization concepts or alternative additives such as cellulose and chitosan is necessary to ensure reproducibility and reliability.

### MRI compatibility

**Fig. 7:**
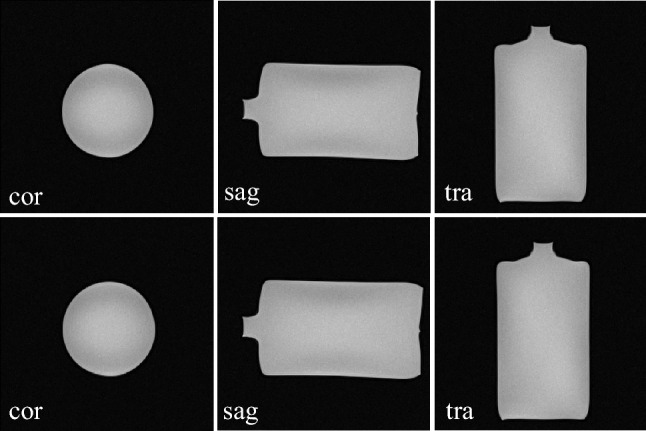
MRI of a standardized MR phantom in coronal (cor), sagittal (sag), and transversal (tra) views to determine the SNR with a SE sequence (TR = 900 ms, TE = 30 ms, slice thickness = 5 mm, matrix 256 × 256, bandwidth = 590 Hz/Pixel). The first row represents the reference imaging (no MU within the scanner room) and the second row represents the imaging during motor motion

The analysis of the averaged reference SNR_r_ = 70.76 and active SNR_a_ = 70.8 over all orientations indicated no reduction in the SNR. No artifacts were visible in MR images (see Fig. 7). Therefore, the MU has no influence on the image quality. If it is installed correctly on the patient table with belts, safety can be ensured.

###  Organ movement

**Fig. 8:**
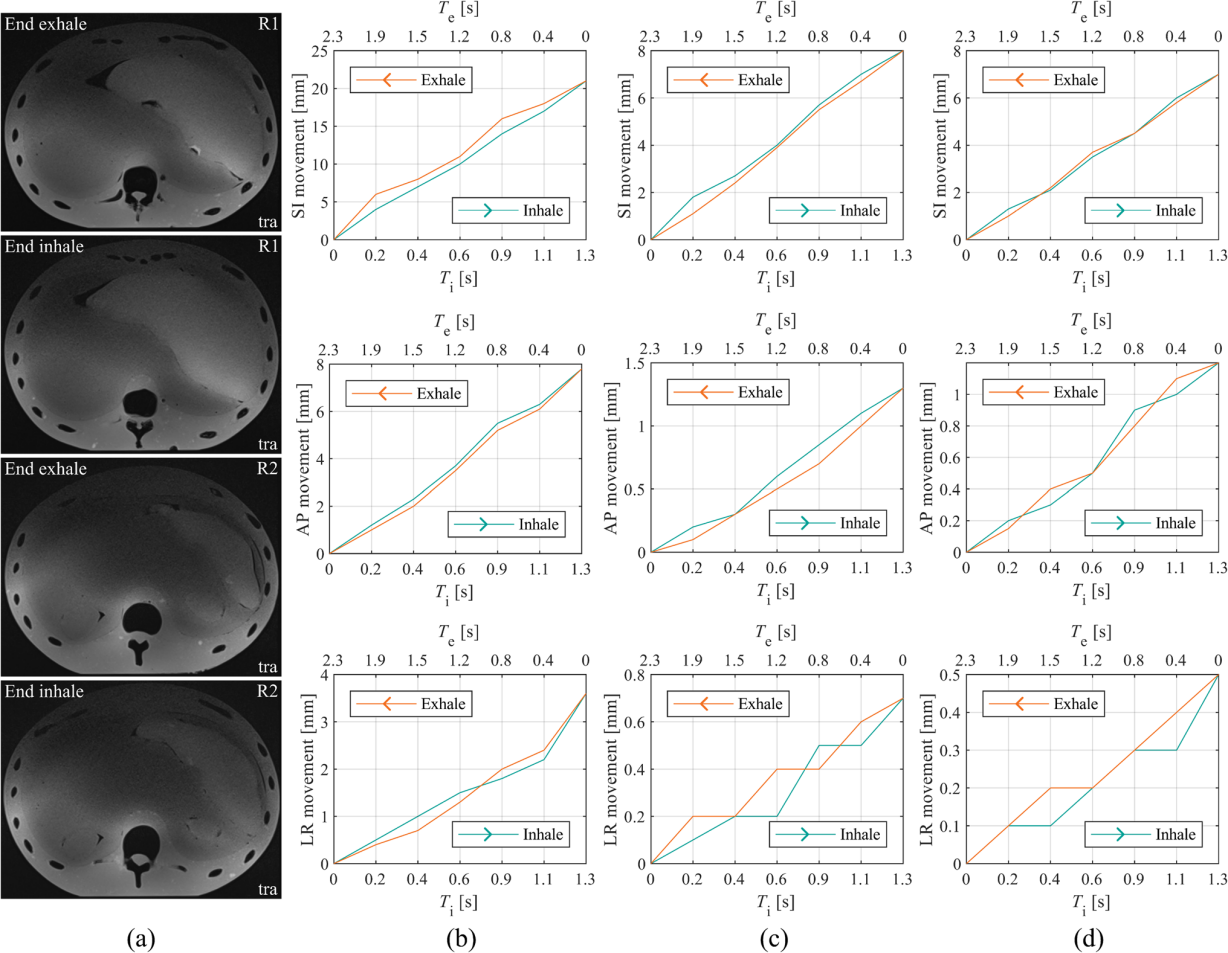
**a** MRI of the APB in transversal (tra) view representing end-exhale and end-inhale phases of the same slice in two regions (R1, R2). Liver (**b**), right kidney (**c**), and left kidney (**d**) motion during inhale and exhale phases in superior-inferior (SI), anterior–posterior (AP) and left–right (LR) direction

The results in Table 3 and Fig. 8 reveal similar APB’s organ movement in comparison with respiratory-triggered human data. The maximum motion occurred from superior to inferior (SI) and the minimum from left to right (RL) such as in patients. Some human breathing pattern indicate inhalation/exhalation hysteresis as partly observed in the moved APB, especially of the liver compartment in the SI direction [21]. The movement became more pronounced, the closer the compartment was located to the DS. During compression, the flexion of the acrylic plates caused additional movements (0.5–1 mm) of the APB.

**Table 3:** Averaged 3D movement of the liver and kidney compartment in comparison with {human data [21]}. Reproducibility error (µ_e_ ± SD) over multiple repositioning

	Liver	Right kidney	Left kidney	Reproducibility *d*_s_ = 0/*d*_s_ = 3
Superior-inferior (SI) [mm]	21 {13}	8 {13}	7 {11}	0 ± 0/0 ± 0
Anterior–posterior (AP) [mm]	7.8 {5.2}	1.3 {6.1}	1.2 {4.4}	0.36 ± 0.07/0.2 ± 0.06
Left–right (LR) [mm]	3.6 {2.1}	0.7 {1.4}	0.5 {1.7}	0.15 ± 0.03/0.23 ± 0.05

The APB’s 3D motion can be performed reproducibly with mean errors in the submillimeter range, which can be caused by the unintended air gaps within the APB. The non-existent SI error resulted from the set imaging slice thickness of 1 mm. Since the human organ motion is not reproducible, these errors are not clinically relevant. The absolute deviation of APB’s motion resulted from the combination of individual variations of the human breathing pattern investigated in [21], which cannot be exactly transferred to the MU.

It should be noted that a realistic organ movement is composed of translation, rotation and deformation, which is not fully considered in the referred references or other in vivo studies so far. Therefore, the simulation of organ motion was fundamentally verified in this work. In future research, the relation between translation-rotation-deformation of each organ (human and phantom) and the influence of the shape of the DS as well as the spatial motion of the rib cage will be investigated.

### Qualitative trend of APB’s properties

Table 4 provides a guide for possible variations achieved in the APB’s properties by changing the concentration of PVA, additional substances and the FTC. The presented values show the property change when increasing the amounts, e.g. doubling the FTC or the amount of sugar. This was the result of the work beforehand to iteratively determine the optimum balance of all properties for the APB. This analysis included the following variations: FTC: 1–4, PVA: 5–15%, NaCl: 1–4%; Sugar: 5–30%; PS: 1–10%.

**Table 4:** Qualitative trend for the MRI and mechanical properties if the FTC and the concentration of the APB’s substances is doubled

Change [%]	T1	T2	*ε*_r_	tan *δ*	*E*
FTC	− 10 to − 30	− 10 to − 50	1 to 10	− 1 to − 10	> 50
PVA	− 30 to − 70	> − 50	1 to 10	10 to 50	> 50
NaCl	− 1 to − 10	− 1 to − 10	1 to 10	> 50	10 to 50
Sugar	− 10 to − 50	− 10 to − 50	− 1 to − 10	− 1 to − 10	30 to 70
PS	− 1 to − 10	5 to 30	1 to 10	30 to 70	− 10 to − 50

For example, if the sugar content in a PVA hydrogel is increased from 15 to 30%, the T1 relaxation time will likely drop about − 10% to − 50% of its previous value, but the Young’s modulus will increase about 30–70% and make it stiffer. The variations originate from the various concentrations and combined substances in the testing pool as well as manufacturing uncertainties.

### Costs

The total costs of the APB are approximately 115 $ (50 $ for the 3D printed ribcage, 40 $ for the 3D printed reusable molds, 25 $ for the PVA and the substances). The summarized costs of the MU are approximately 360 $ (85 $ for the acrylic plates, 55 $ for the 3D-printed mechanical parts & screws, 120 $ for the drive components, 55 $ for the microcontroller & electronics, 45 $ for the aluminum housing).

The given indication excludes the working hours and laboratory equipment (3D-printers, laser structuring system, CNC machine, etc.).

## Conclusion

This work presents a novel modularizable and low-cost PVA-C based abdominal phantom to mimic fundamental organ movement. It can be applied during needle-based punctures within an MRI environment without any image quality losses. The respiration pattern can be set with time and distance of inhalation/exhalation individually. According to the experiments, the phantom’s characteristics showed an agreement of relaxation times, dielectricity and elasticity in comparison with the respective human tissue, enabling a wide range of feasible applications. It was resistant against mold and resealable after punctures, but could not guarantee stable properties for long-term use.

Future research and development will be focused on analyzing the translation-rotation-deformation ratio of each organ (human and phantom) and the chemical composition of APB to ensure long-term durability not only for iMRI, but also computer tomography-based and ultrasound-based interventions. The mold resistance according to ASTM G21-15 will be analyzed in more detail. Furthermore, different shaped DS can enable optimized spatial motion behavior of organs. This phantom improves the development and safety testing of medical devices in addition to or as an alternative to in vivo studies as well as a versatile training environment for physicians.
